# Bis(2,2′-bipyridil)Copper(II) Chloride Complex: Tyrosinase Biomimetic Catalyst or Redox Mediator?

**DOI:** 10.3390/ma14010113

**Published:** 2020-12-29

**Authors:** Milan Sýs, Atripan Mukherjee, Granit Jashari, Vojtěch Adam, Amir M. Ashrafi, Miroslav Novák, Lukáš Richtera

**Affiliations:** 1 Department of Analytical Chemistry, Faculty of Chemical Technology, University of Pardubice, Studentská 573, 532 10 Pardubice, Czech Republic; milan.sys@upce.cz (M.S.); granit.jashari@student.upce.cz (G.J.); 2 Department of Chemistry and Biochemistry, Mendel University in Brno, CZ-613 00 Brno, Czech Republic; xmukherj@mendelu.cz (A.M.); vojtech.adam@mendelu.cz (V.A.); ashrafi@mendelu.cz (A.M.A.); 3 Central European Institute of Technology, Brno University of Technology, 612 00 Brno, Czech Republic; 4 Institute of Chemistry and Technology of Macromolecular Materials, Faculty of Chemical Technology, University of Pardubice, Studentská 573, 532 10 Pardubice, Czech Republic; miroslav.novak@upce.cz

**Keywords:** electrochemical biomimetic devices, bis(2,2′-bipyridil)copper(II) chloride complex, flow injection analysis, neurotransmitters

## Abstract

In this article, construction of amperometric sensor(s) based on screen-printed carbon electrodes covered by thin layers of two types of carbon nanomaterials serving as amplifiers, and containing [Cu(bipy)_2_Cl]Cl∙5H_2_O complex is reported. Their performance and biomimetic activity towards two selected neurotransmitters (dopamine and serotonin) was studied mainly using flow injection analysis (FIA). The important parameters of FIA such as working potential, flow rate, and pH were optimized. The mechanism of the catalytic activity is explained and experimentally confirmed. It reveals that presence of hydrogen peroxide plays a crucial role which leads to answer the title question: can presented complex really be considered as a tyrosinase biomimetic catalyst or only as a redox mediator?

## 1. Introduction

Metal complexes are defined as a central metal atom or ions, named as the coordination center, surrounded by bound molecules or ions, known as ligands. Metal complexes have found broad application in different fields, particularly in medicine where cisplatin (cis-[PtCl_2_(NH_3_)_2_]) is one of the world’s best-selling anticancer drug. Moreover, phosphine ligand compounds containing gold, silver, and copper also have anti-cancer properties [1,2]. Furthermore, some families of metal complexes showed promising properties to be used in photodynamic therapy [3]. In fact, transition metals play the key role in several biological processes including electron transfer, and catalysis as the active site of the proteins and enzymes [4]. Due to their catalytic properties, the metal complexes were also used in modification of the electrodes applied in electroanalytical determinations, where reducing of the overpotential required for the reduction or oxidation of the target analyte is desired [5]. Bearing in mind that electroanalytical methods are featured with superior sensitivity, simplicity, minimal sample pretreatment requirement as well as being inexpensive, the further progress to address the existent challenges in electroanalysis is highly demanded. Therefore, the electrocatalysis, which is one of the most important approaches for improving the sensitivity as well as the selectivity, is of great importance in the electroanalysis [6]. In fact, the metal complexes function as fast electron transfer mediators. Hence, an analyte which is reduced (oxidized) slowly at the bare electrode at high magnitude of the cathodic (anodic) potential, undergoes electrochemical reduction (oxidation) at lower potential magnitude resulting in higher sensitivity and better selectivity [7,8].

However, it must be noticed that the redox overpotential of other compounds presenting in the matrix may also be decreased which may bring about interfering effect. To overcome this problem, usually the protective membranes are used which eliminate the interferents based on their charge or size, that consequently improves the selectivity [9,10]. The membrane also protects the modified layer from being washed of the electrode surface. Besides, the incorporation of the complex into the carbon materials with less water solubility also increases the stability of the modified layer on the electrode surface [11,12,13].

Among the others, the copper complexes were also used as the mediators in electroanalysis, which were associated with improved sensitivity, selectivity and shorter response time [14,15,16]. Because of its application in a broad field such as pharmaceutical, textile and food manufacturing industries [17], medical diagnosis [18], water treatment [19], the determination of hydrogen peroxide (further only H_2_O_2_) is important.

In biosensing, the H_2_O_2_ correlated to the target analyte, especially for the indirect determination of many compounds representing substrates of numerous enzymes, called as oxidoreductases, having a flavin mononucleotide (FMN) as cofactor. A typical example is a glucose oxidase from *Aspergillus niger* (EC 1.1. 3.4) [20]. The H_2_O_2_ undergoes irreversible redox reactions providing broad cathodic or anodic peak occurring at high potentials. To improve the selectivity of the amperometric detection of H_2_O_2_, transducer surfaces are modified with substances that have catalytic (mediating) activity including: (I) transition metal ions, their oxides or complexes [14,21,22] (II) organic dyes, especially Prussian blue [23] or iron phtalocyanine [24], and (III) nanomaterials [25].

**Figure 1:**
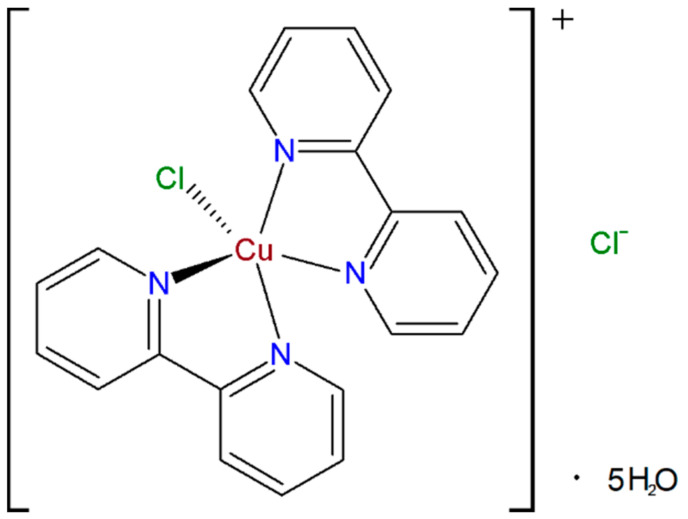
Chemical structure of [Cu(bipy)_2_Cl]Cl∙5H_2_O complex.

Even though this work was not aimed to develop a method for the selective and sensitive determination of H_2_O_2_ using the bis(2,2ʹ-bipyridil)copper(II) chloride ([Cu(bipy)_2_Cl]Cl 5H_2_O) complex (CBCC) (Figure 1) as the mediator and Nafion^®^ as the protective layer that avoids washing the complex of the electrode surface. Nevertheless, important analytical parameters of H_2_O_2_ sensing are also included.

Herein, it is necessary to mention an application of the (CBCC) in amperometric determination of three representatives of diphenols (catechol, dopamine, and L-3,4-dihydroxyphenylalanine) was reported in 2003 [26]. Authors stated the fact that this copper complex mimics the tyrosinase (catecholase) activity and could be used for dopamine determination. Unfortunately, they did not study a cresolase activity toward monophenols.

Assuming that the copper atom in the above-mentioned complex is kept in Cu^+^ form at constant negative potential, an addition of H_2_O_2_ will oxidize the Cu^+^ to Cu^2+^ form which will be reduced electrochemically to obtain reduction current response. Moreover, the H_2_O_2_ represents a strong oxidizing agent which may be able to chemically oxidize the dopamine or other neurotransmitters to corresponding quinones.

The aim of this work is to reveal whether the present copper complex can be really considered as a tyrosinase biomimetic catalyst or only as a redox mediator. This includes improvements in the sensitivity of hydrogen peroxide detection (amperometric transducer for oxidoreductases-based biosensors) where two different types of carbon nanomaterials (CNMs) were investigated as potential amplifiers of the measured current signal. Regarding the last one, a flow injection analysis system utilizing the wall-jet configured flow cell with an incorporated planar biomimetic sensor was proposed where a special attention was paid to the selectivity towards dopamine and serotonin.

## 2. Materials and Methods

### 2.1. Chemicals and Instrumentation

Graphene oxide (GO) of resistivity ≤0.30 Ω cm and specific surface area 400–1000 m^2^ g^−1^) from ACS Material, LLC (Medford, OR, USA) and multi-walled carbon nanotubes (MWCNTs) of diameter 10–30 nm, length 5–15 μm, and specific surface area 40–300 m^2^ g^−1^ from Shenzhen Nanotech Port Co., Ltd. (Shenzhen, China) were investigated as suitable amplifiers of current signal.

Chemicals, such as tyrosinase (EC 1.14.18.1) lyophilized solid powder from mushroom (*Agaricus bisporus*) of enzyme activity ≥1000 U mg^−1^, (*N,N*-dimethylformamide (DMF), 5% (*w*/*w*) Nafion^®^ in mixture of 55% (*v*/*v*) ethanol, 25% ammonium solution, 30% hydrogen peroxide, ≥99% sodium carbonate, 98% sodium borohydride, sodium phosphate dibasic, potassium dihydrogen phosphate, potassium chloride, serotonin hydrochloride, and dopamine hydrochloride (all in *w*/*w*), were purchased from Merck KGaA (Darmstadt, Germany). The deionized water (18.2 MΩ cm) used for preparation of 0.1 mol L^−1^ phosphate buffer (PB) with pH of 7.5 was at first double-distilled by an Aqua Osmotic 02 (Aqua Osmotic, Tisnov, Czech Republic) and then deionized by using a Millipore RG (Milli-Q water, Millipore Corp., Billerica, MA, USA). A stock solution of 0.1 mol L^−1^ hydrogen peroxide was always freshly prepared and used for electrochemical experiments.

In 50 mL glass cell, most voltammetric and amperometric (in batch configuration) measurements were performed in either pure 0.1 mol L^−1^ phosphate buffer of pH 7.5 with content of 0.05 mol L^−1^ KCl or, in addition, with the presence of 1 mmol L^−1^ (CBCC). Chloride anions were essential for the proper function of commercially screen-printed carbon electrodes (SPCEs; type DRP-C110 and DRP-C150, both from Metrohm DropSens, Oviedo, Spain) due to using the silver-only pseudoreferent electrode.

These measurements were carried in a conventional three-electrode arrangement consisting always on of tested electrodes, a reference silver/silver chloride electrode with 3 mol L^−1^ KCl as salt bridge from Metrohm (Herisau, Switzerland), and platinum sheet, as auxiliary electrode, from Elektrochemické detektory, s. r. o. (Turnov, Czech Republic).

Flow injection analysis (FIA) configuration consisted of a multi-channel peristaltic pump MINIPULS 3 from Gilson (Middleton, WI, USA), a Rheodyne automatic six-position dosing valve from IDEX Health & Science (Wertheim, Germany), one of tested sensors inserted into the wall-jet flow cell from above-mentioned Metrohm DropSens company. The 0.1 mol L^−1^ PB (pH 7.5) containing 0.05 mol L^−1^ KCl and 500 µmol L^−1^ H_2_O_2_ was used as flowing carrier solution (FCS). All electrodes were connected to potentiostat/galvanostat Autolab PGSTAT101 operated via the NOVA 1.11 software from the AUTOLAB, Metrohm Autolab B.V. (Utrecht, The Netherlands). Spectrophotometric measurements were carried out using UV-Vis spectrophotometer UV2450 from Shimadzu (Kyoto, Japan). Scanning electron microscopy (SEM) used for characterization of all tested sensors was performed at VEGA3 SBU (TESCAN, Czech Republic).

### 2.2. Synthesis of Bis(2,2ʹ-bipyridil)Copper(II) Chloride Complex [Cu(bipy)_2_Cl]Cl∙5H_2_O

The (CBCC) was synthesized according to the reported procedure [26,27,28]. The solution of CuCl_2_·2H_2_O (0.25 g, 1.47 mmol) in EtOH (10 mL) was added dropwise to the stirred EtOH solution (10 mL) of 2,2′-bipyridine (0.46 g, 2.94 mmol) at room temperature. The reaction mixture was stirred for 4 h. After that, the cooling of the reaction mixture to −20 °C afforded (CBCC) (0.42 g, 53%) as blue crystalline material during 24 h. All analytical data were in accordance with those reported in literature [26,27,28]. Moreover, the structure was verified by evaluation of full scan mass positive-ion electrospray mass spectrum, as shown in Figure S1 (Supplementary file).

### 2.3. Chemical Reduction of Graphene Oxide

Reduced graphene oxide (rGO) was prepared by well-established and optimized method used for standard experiments [29]. Amount of 1.0 g graphene oxide (GO) was dispersed in 1 L deionized water to give a colloidal solution. The pH value of GO dispersion was adjusted to 9–10 by addition of 5% solution of Na_2_CO_3_. The mixture was heated up to 80 °C and then 800 mg of Na[BH_4_] was added as a reducing agent. The temperature was maintained at 80 °C for 1 h. Final reduced graphene oxide (rGO) was collected and washed three times with deionized water.

### 2.4. Preparation of Amperometric Sensors Utilizing [Cu(bipy)_2_Cl]Cl∙5H_2_O Complex

In order to select optimum amperometric transducer, four different carbon-based electrode substrates were tested, such as unmodified glassy carbon, rGO, MWCNTs, and carbon ink (SPCE). Surface of solid glassy carbon electrode (GCE) having diameter of 3.0 mm from already mentioned Metrohm (Herisau, Switzerland) was renovated on polishing pad with presence of wet Al_2_O_3_ powder with particle size of 1.0 μm from (Metrohm, Herisau, Switzerland) for 30 s and subsequently placed in an ultrasonic bath for 5 min. After subsequent rinsing of the surface by stream of deionized water, the GCE was ready for surface modification by CNMs. Dispersions of MWCNTs and rGO in DMF (2 mg mL^−1^) had to be homogenized by ultrasound at laboratory temperature for 60 min. The modification is consisted of applying of 20 μL (10 μL for SPCE; 4 times of 2.5 μL) corresponding dispersion and allowed to be dried at room temperature for 24 h. Due to the high wettability of the dispersions on the surface of SPCEs, they had to be applied in four consecutive steps of 2.5 µL, known as multiple layering.

A volume of 10 mL of stock solution 4 mg mL^−1^ (CBCC) in DMF was prepared in small glass vial. Then, 100 µL of the corresponding complex solution was mixed with 50 µL of 5% Nafion^®^ (neutralized by 8% ammonium solution) in plastic Eppendorf vials. Finally, a volume of 20 µL of resulting complex membrane solution (4 times of 5 μL in case of SPCE) was applied onto prepared electrode surfaces and was left to be dried at laboratory conditions for 2 h. Before each electrochemical measurement, the freshly prepared sensors had to be kept in buffer solution for 20 min to be Nafion^®^ hydrated.

### 2.5. Methods

Spectrophotometric measurements in ultraviolet and visible region were performed in 1 cm quartz cuvette from Fisher Scientific (Pardubice, Czech Republic) in the range of wavelengths from 200 to 800 nm at scanning speed of 0.5 nm s^−1^. The cuvette contained 500 µmol L^−1^ H_2_O_2_ and 200 µmol L^−1^ randomly chosen neurotransmitters in 0.1 mol L^−1^ PB (pH 7.0).

Voltammetric measurements were carried out in 0.1 mol L^−1^ PB (pH 7.5) containing 0.05 mol L^−1^ KCl using cyclic voltammetry in potential range from −0.6 to +0.6 V, potential step of 2.5 mV, and scan rates (*ν*) varying from 20 to 500 m Vs^−1^. Amperometric measurements in batch configuration was performed in 0.1 mol L^−1^ PB (pH 7.5) containing 0.05 mol L^−1^ KCl at detection potential ranging from −0.4 to +0.1 V and stirring speed of 400 rpm. In contrast, 0.1 mol L^−1^ PB (pH 7.0) containing 0.05 mol L^−1^ KCl and 500 µmol L^−1^ H_2_O_2_ was used as flowing carrier solution (FCS) in FIA in the wall-jet configuration at flow rate of 1 mL min^−1^ and detection potential of −0.3 V. Otherwise, all essential changes in the working parameters are described in the legends of the corresponding figures.

## 3. Results

### 3.1. Structures of CNMs Layers Covered with Nafion^®^ Membrane Containing [Cu(bipy)_2_Cl]Cl 5H_2_O

**Figure 2:**
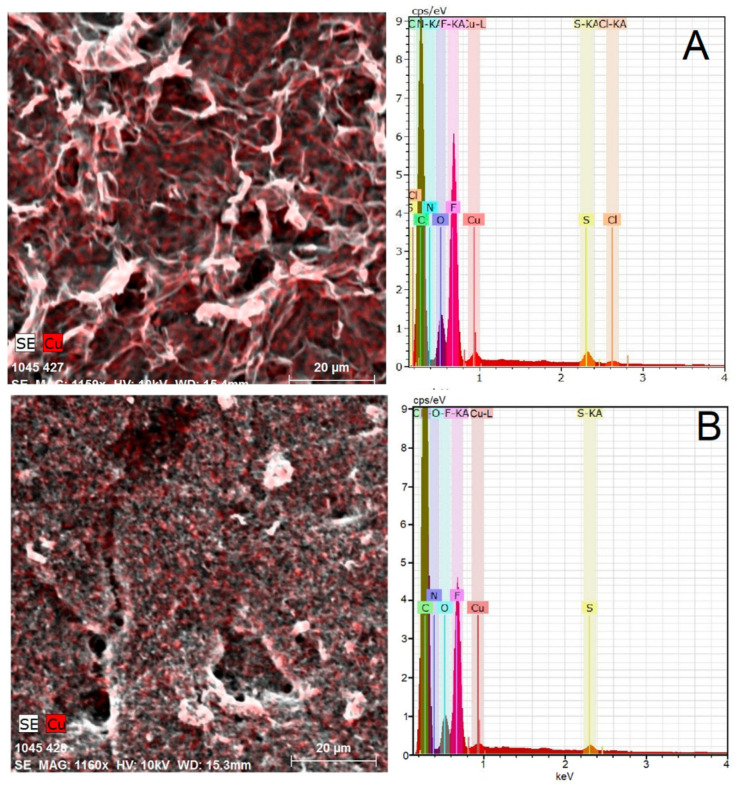
Elemental mapping of rGO (**A**) and MWCNTs (**B**) Elemental mapping of MWCNTs layers covered by Nafion^®^ membrane containing the [Cu(bipy)_2_Cl]Cl∙5H_2_O complex using SEM-EDX spectroscopy.

MWCNTs and rGO can be defined as electrically conductive nanomaterials with large specific surface areas. Nowadays, they are widely used in the development of biomimetic sensors [30]. A scanning electron microscopy (SEM) with energy-dispersive X-ray spectroscopy (EDX) were used as a routine tool for characterization of electrode surfaces morphology and elemental mapping. The surfaces of the immobilized rGO and MWCNTs layers covered with Nafion^®^ membrane containing (CBCC) are not smooth as in the case of bare GCE. At magnification 1200×, it was found that a typical planar configuration of rGO is the main reason that rGO layer resembles a coarse cloth (Figure 2A) and MWCNTs create a multilayer carpet with the presence of irregular skeins of various sizes (Figure 2B). The elemental mapping revealed a homogeneous distribution of (CBCC) molecules. Unfortunately, the molecules of the complex form a ring in the Nafion^®^ membrane on the surface of the bare GCE, visible to the naked eye (Figure S2). EDX spectra obtained confirm the presence of several elements, namely F and S from Nafion^®^ (sulfonated tetrafluoroethylene based fluoropolymer-copolymer), Cu, N, and Cl from (CBCC).

### 3.2. Effect of Carbon Nanomaterials

In order to find the optimum amount of CNMs onto GCE surface, six different dispersions (0.5, 1.0, 1.5, 2.0, 2.5, and 3.0 mg mL^−1^) of CNMs (MWCNTs or rGO) in DMF were prepared and always a volume of 20 μL of the corresponding dispersion was applied for immobilization onto the electrode surface. Using cyclic voltammetry, 1.0 mmol L^−1^ CBCC in 0.1 mol L^−1^ PB (pH 7.5) was investigated at the resulting transducers differing in CNMs amounts. In comparison between using MWCNTs and rGO (both having 40 µg CNMs), no significant difference was found. The presence of CNMs caused the ratio of the peak currents (*I*_p_^a^/*I*_p_^c^) to be equal to one. Moreover, several times higher peak heights were obtained at GCE/CNMs in contrast with bare electrodes (see Figure S3). Regarding the effect of CNMs amount, reversible peak current signals of CBCC increased with higher CNMs content (20–40 µg) onto GCE surface. Higher amounts than 40 µg CNMs caused a slight decrease in peak current responses, and therefore the dispersion of 2.0 mg mL^−1^ CNMs in DMF was chosen as the optimum.

### 3.3. Effect of Nafion^®^ Layer

Generally, Nafion^®^ represents a micellar polymer that allows for the channel-assisted diffusion of small cations. Due to presence of sulfonic acid side chains, [Cu(bipy)_2_Cl]^+^ cations are probably aggregated in the micelle centers. For this reason, the Nafion^®^ was preferred over other polymers. Figure S4 presents a comparison of cyclic voltammograms of CBCC present in the bulk solution and CBCC immobilized in the Nafion^®^ membrane. It seems that used perfluorinated resin naturally blocks the mass transfer. This phenomenon is more visible at high scanning speeds. The peak-to-peak separation (Δ*E*_p_) of CBCC increased with the higher amount of Nafion^®^ onto GCE surface. Within Nafion^®^ content immobilization, it was necessary to find a compromise between the current response and membrane stability, to prevent the elution of complex molecules under flow conditions.

Five membrane solutions differing in the Nafion^®^ content (*w*/*w*) (0.5, 1.0, 1.5, 2.0, 2.5, and 3.0%) were investigated using repetitive cyclic voltammetry in 0.1 mol L^−1^ PB (pH 7.5) at GCE/CBCC-Nafion^®^ electrodes always having the same amount of CBCC (80 µg). The stable redox current signals were obtained for electrodes with the Nafion^®^ content higher than 2% (*w*/*w*), and therefore the 2.5% Nafion^®^ content (*w*/*w*) was considered as the optimum.

### 3.4. Effect of [Cu(bipy)_2_Cl]Cl∙5H_2_O Content in Nafion^®^ Membrane

In order to find out the effect of the (CBCC) content, 10 mL stock solutions containing 2, 4, 6, 8, and 10 mg mL^−1^ (CBCC) in DMF were prepared in small glass vials. Assuming that always 100 µL CBCC solution was mixed with 50 µL of 5% (*w*/*w*) Nafion^®^ and then 20 µL of the resulting membrane solution was applied onto GCE surface, the total amount of CBCC was calculated. A comparison of GCE/CBCC-Nafion^®^ electrodes in amperometric detection of 100 µmol L^−1^ H_2_O_2_ showed that higher content than 60 µg of CBCC did not provide any significant improvement in reduction current response. Therefore, 60 µg of CBCC (4 mg mL^−1^ CBCC in DMF) was chosen as the optimum.

### 3.5. Electrochemical Comparison of Constructed Amperometric Sensors

Electrochemical characterisation of (CBCC) present in the Nafion^®^ membrane (>50 μg) covering the surfaces of GCE, GCE/MWCNTs, and GCE/rGO was performed using cyclic voltammetry measurements focused on redox couple of Cu(II)/Cu(I) formation. Since the GCE serves only as an electrical conductor, a similar electrochemical behaviour can be expected for SPCEs covered with the corresponding CNMs (SPCE/MWCNTs/CBCC-Nafion^®^ or SPCE/rGO/CBCC-Nafion^®^). Because the mentioned sensors differed in their surface areas, the current responses obtained during all electrochemical measurements are presented as current density (*j*) in unit of µA mm^−2^.

**Figure 3:**
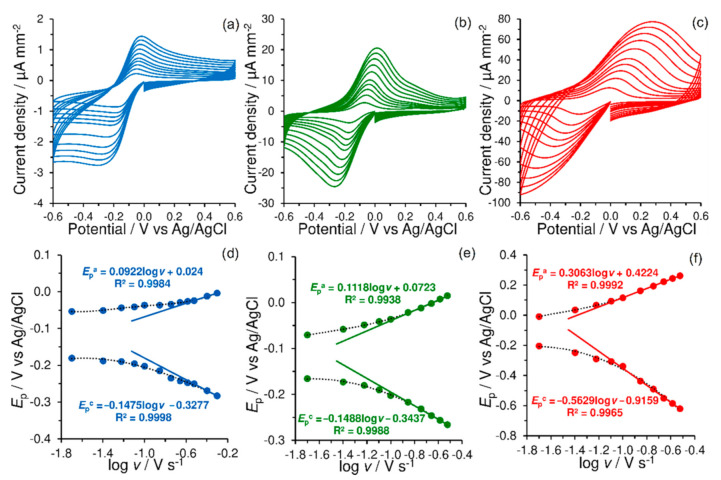
Cyclic voltammograms of 0.1 mol L^−1^ PB (pH 7.5) at GCE/CBCC-Nafion^®^ (**a**), GCE/MWCNTs/CBCC-Nafion^®^ (**b**), and GCE/rGO/CBCC-Nafion^®^ (**c**) obtained at scan rate of 20, 40, 60, 80, 100, 140, 180, 220, 260, and 300 mV s^−1^ with the corresponding trumpet plots (**d**–**f**).

As shown in Figure 3, [Cu(bipy)_2_Cl]Cl∙5H_2_O complex immobilized inside the Nafion^®^ membrane provided typical Cu^2+^/Cu^+^ redox couple at all tested sensors in 0.1 mol L^−1^ PB (pH 7.5). The CNMs presence significantly amplified the measured current signal due to their specific surface areas. This means that lower values of detection and quantification limits will be achieved using this simple modification.

Generally, increasing the scan rate (*ν*) usually reveals the kinetics of electron transfer and coupled reactions through representations, known as trumpet plots (peak potentials against log*ν*). The corresponding graphs are shown below in Figure 3. Because the copper complex represents a water-soluble substance that can move to the solution only by washing out from the membrane, data obtained can be analyzed using a procedure introduced by Laviron [31]. Values of transfer coefficient (α) for each electrode were calculated as 0.38 for GCE/CBCC-Nafion^®^, 0.43 for GCE/MWCNTs/CBCC-Nafion^®^ and 0.35 for GCE/rGO/CBCC-Nafion^®^. This kinetic parameter is commonly used to describe the symmetry between the forward and reverse electron transfer steps. Considering the α values, it can be concluded that the oxidation reaction is favored.

### 3.6. Effect of the Applied Potential

The detection potential plays a fundamental role in biomimetic sensing, especially in case of redox mediators, because the applying of rather high negative constant potential can reduce the Cu^2+^ (oxidized form) in the present complex to Cu^+^ (reduced form). It is the reason why an injection of H_2_O_2_ provides a negative current response (chemical oxidation of Cu^+^ to Cu^2+^ by H_2_O_2_ and the subsequent electrochemical reduction of the resulting Cu^2+^ to Cu^+^ with participation of one electron) [32]. A negligible current response will be obtained if a dopamine injection follows because the dopamine cannot be chemically oxidized by presented H_2_O_2_. The copper in the complex is electrochemically kept at its oxidation state Cu^2+^ by applying an adequate potential. In the presence of H_2_O_2_, two molecules of the copper complex join by oxygen bridges to form an active center similar to that of tyrosinase enzyme [26], which is able to catalyze the oxidation of phenolic substances to the corresponding quinones. These quinoid compounds represent electroactive species which can be amperometrically detected.

On the other hand, setting a high positive detection potential is able to keep the copper in its oxidation form Cu^2+^ and causes a negligible current response for injection of H_2_O_2_, and in addition, electrochemically oxidizes the dopamine to dopamine-*o*-quinone with participation of two electrons and protons which undergo the subsequent polymeric reaction to form a polydopamine [33].

Hence, it can be assumed that the optimum value of detection potential (half-wave potential) should be found to achieve the proper function of (CBCC)-based sensor as tyrosinase biomimetic sensor for amperometric determination of neurotransmitters (phenolic compounds).

**Figure 4:**
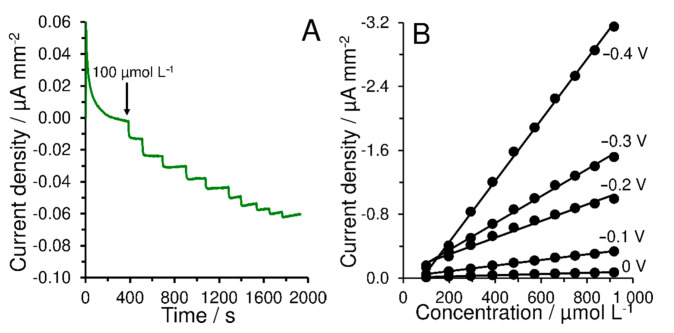
Typical amperometric response of GCE/MWCNTs/CBCC-Nafion^®^ to different concentrations of H_2_O_2_ (100–920 µmol L^−1^) obtained at 0.0 V vs Ag/AgCl (**A**) and calibration curves obtained at different applied potentials (**B**). Each measurement was performed in 0.1 mol L^−1^ PB (pH 7.0) at stirring speed of 400 rpm.

Figure 4 shows an amperometric response for injection of H_2_O_2_ (batch configuration) obtained at 0.0 V with the GCE covered by thin layer of MWCNTs and Nafion^®^ membrane containing the copper complex (GCE/MWCNTs/CBCC-Nafion^®^) and five calibration curves obtained using this sensor at different values of applied potential. The sensitivity increased with setting more negative applied potential, namely −0.00008, −0.0004, −0.001, −0.0017, and −0.0038 µA mm^−2^ µmol^−1^ L for 0.0, −0.1, −0.2, −0.3, and −0.4 V, respectively.

If current response of Cu^2+^ reduction (forming from Cu^+^ oxidized by H_2_O_2_) is sensed at different applied potentials (from +0.1 to −0.4 V), the maximum current response will be obtained at −0.4 V. However, the applied potential of −0.3 V was preferred as optimum due to satisfying repeatability caused by low noise of current signal. This fact was confirmed using amperometric measurements in the batch configuration and FIA, as shown in Figure S5. It can be assumed that the ability to perform electrochemical detection at 0.0 V allows GCE/MWCNTs/CBCC-Nafion^®^ to be used as a suitable amperometric transducer for the development of highly selective biosensors useful in clinical analysis.

According to the proposed mechanism for phenolic compounds oxidation for the sensor modified with CBCC [26], it is possible to assume that the cooper has to be occurred in Cu^2+^ form to be able to catalyze the oxidation of phenolic compounds (neurotransmitters). However, the detection potential must be negative in order to be able to electrochemically reduce the oxidation products formed (dopamine-*o*-quinone [34] and tryptamine-4,5-dione [35]) but not as much to reduce all of the Cu^2+^ to the Cu^+^ form. Hence, an effect of several applied potentials (from −0.20 to +0.01 V) on dopamine (500 µmol L^−1^) current response was investigated using amperometric measurements in the batch configuration performed in 0.1 mol L^−1^ PB containing 0.05 mol L^−1^ KCl and 200 µmol L^−1^ H_2_O_2_ (pH 7.0). The maximum current yield was obtained at applied potential of −0.1 V, and therefore this value was chosen as the optimum. Moreover, an undesirable phenomenon (significant reduction in current response after each analyte addition) was observed which can be explained by the formation of polydopamine which covers the surface of the GCE/MWCNTs/CBCC-Nafion^®^ electrode. Fortunately, this problem could be solved by using fast flow rate of flowing carrier in the FIA which will wash away the generated products of electrochemical detection.

### 3.7. Effect of Flowing Carrier pH

FIA measurements of five consecutive injections of 100 µmol L^−1^ H_2_O_2_ were carried out at detection potential of −0.3 V in 0.1 mol L^−1^ PBs with 0.05 mol L^−1^ KCl differing in pH value from 5 to 9. The investigation of pH effect on current response showed that SPCE/MWCNTs/CBCC-Nafion^®^ provides the optimum current response at the pH of 7.0. This observation is in agreement with the data reported previously [26]. It can be assumed that catalytic activity of CBCC is related to stability of nitrogen-coordinated bounds of copper atom. In acidic environment, a protonation of nitrogen atoms causes the release of copper ions into bulk solution. In contrast, in the alkaline environment, Cu^2+^ cations can undergo to precipitation reaction to form Cu(OH)_2_.

### 3.8. Effect of Flow Rate

The flow rate can be included among the key parameters affecting the sensitivity, represented by slope (*k*) of the calibration curve, because it defines the time duration over which H_2_O_2_ or neurotransmitters are in the column where reduction of Cu^+^ cations by H_2_O_2_ or probable oxidation of neurotransmitters catalysed by Cu^2+^ cations occurs. At the applied potential of −0.3 V, flow rate was varied as 0.4, 0.6, 0.8, 1.0, 1.2, and 1.4 mL min^−1^. Herein, it was found that current response (peak height) slightly increased with increasing the flow rate up to 1.0 mL min^−1^. Because setting higher values of flow rate did not bring any significant improvement in the current response, the flow rate of 1.0 mL min^−1^ was chosen as optimum.

### 3.9. Amperometric Detection of Hydrogen Peroxide at [Cu(bipy)_2_Cl]Cl∙5H_2_O Complex-Based Sensors

A commercial SCPE (type DRP-C150) covered by a thin layer of Nafion^®^ membrane containing the [Cu(bipy)_2_Cl]Cl∙5H_2_O complex (SPCE/CBCC-Nafion^®^) together with SPCE/MWCNTs/CBCC-Nafion^®^ and SPCE/rGO/CBCC-Nafion^®^ were compared in determination of H_2_O_2_ using FIA at optimum conditions to find out whether any presence of CNMs can improve the H_2_O_2_ electro-sensing.

**Figure 5:**
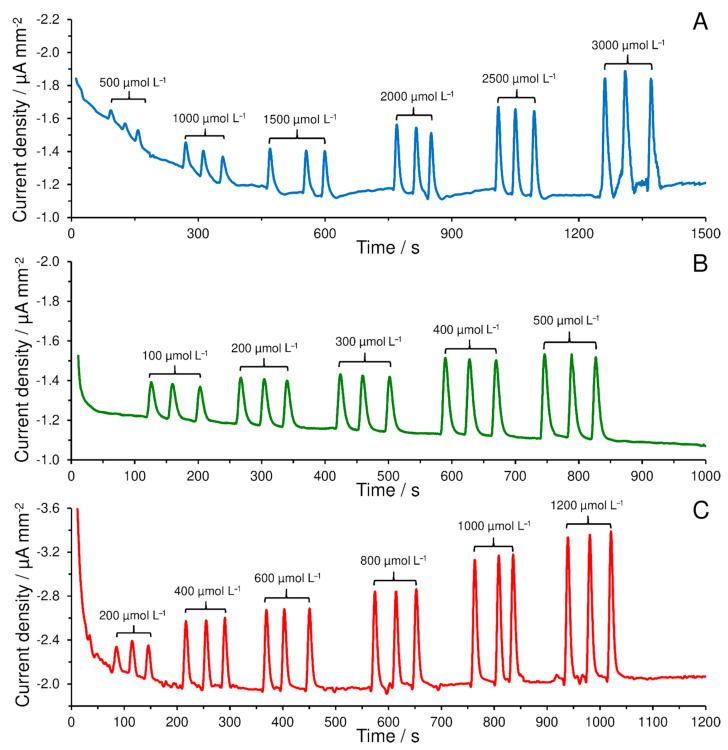
FIA records of H_2_O_2_ obtained using SPCE/CBCC-Nafion^®^ (**A**), SPCE/MWCNTs/CBCC-Nafion^®^ (**B**), and SPCE/rGO/CBCC-Nafion^®^ (**C**) at flow rate of 1 mL min^−1^ of 0.1 mol L^−1^ PB with 0.05 mol L^−1^ KCl content (pH 7.0) and applied potential −0.3 V.

At first glance, it is clear that the presence of CNMs had a fundamental influence on the overall sensitivity. For a concentration range from 0.5 to 3.0 mmol L^−1^ H_2_O_2_. (Figure 5A), a calibration curve given by an equation of linear regression *j* (µA mm^−2^) = −0.0002*c* (µmol L^−1^) − 0.0873 with coefficient of determination (*R*^2^) 0.9919 using SPCE covered only by Nafion^®^ membrane with the copper complex (SPCE/CBCC-Nafion^®^) has been achieved. Amperometric sensors employing CNMs provided many times higher sensitivity than the one previously mentioned (Figure 5B and Figure S4). Linear ranges for evidently lower concentrations were found (100–500 and 200–1200 µmol L^−1^ H_2_O_2_) that are described by regression equations, namely *j* (µA mm^−2^) = −0.0006*c* (µmol L^−1^) − 0.1036 with *R*^2^ = 0.9950 and *j* (µA mm^−2^) = −0.001*c* (µmol L^−1^) − 0.1328 with *R*^2^ = 0.9924 for SPCE/MWCNTs/CBCC-Nafion^®^ and SPCE/rGO/CBCC-Nafion^®^, respectively.

Values of detection and quantification limits were calculated as three and ten times of the standard deviation (*s*) of three replicate injections (100 µmol L^−1^ H_2_O_2_) divided by the slopes of the corresponding regressions (*k*), respectively. All analytical parameters are demonstrated in Table 1. The analytical parameters were slightly better for rGO, and therefore the sensor based on rGO was chosen for following studies.

### 3.10. Optimisation of Hydrogen Peroxide Content in Flowing Carrier for Neurotransmitters Electro-Sensing

Figure S6 shows the results obtained in the FIA experiments carried out in the absence of H_2_O_2_ at −0.1 V. At first glance, it is evident that the presence of H_2_O_2_ in the flowing carrier solution is necessary for the oxidation catalysis. It seems that the molecule of H_2_O_2_ probably helps to connect two molecules of the cooper complex by oxygen bridges to form an active site like in the case of the oxy-tyrosinase form [36] which has the ability to catalyze the oxidation of phenolic substances.

Five different contents of H_2_O_2_ (0, 50, 200, 500, and 1000 µmol L^−1^) in the flowing carrier were used and the current responses for the three injections of 100 µmol L^−1^ dopamine were monitored. In comparison with amperometry in the batch configuration, where the 200 µmol L^−1^ H_2_O_2_ content was sufficient (should be compared with 150 µmol L^−1^ H_2_O_2_ recommended in the literature [26]), the presence of 500 µmol L^−1^ H_2_O_2_ was considered as optimum for FIA because the higher H_2_O_2_ content did not cause any significant improvement.

### 3.11. Analytical Performance of FIA with Integrated Amperometric Sensor

Two typical examples of neurotransmitters, namely dopamine and serotonin, were deliberately selected to determine if the CBCC is capable of mimicking the cresolase and catecholase activities. Unlike serotonin that has only one hydroxyl group onto an indole ring, the dopamine is included among catecholamines having two hydroxyl groups substituted onto a benzene ring.

**Figure 6:**
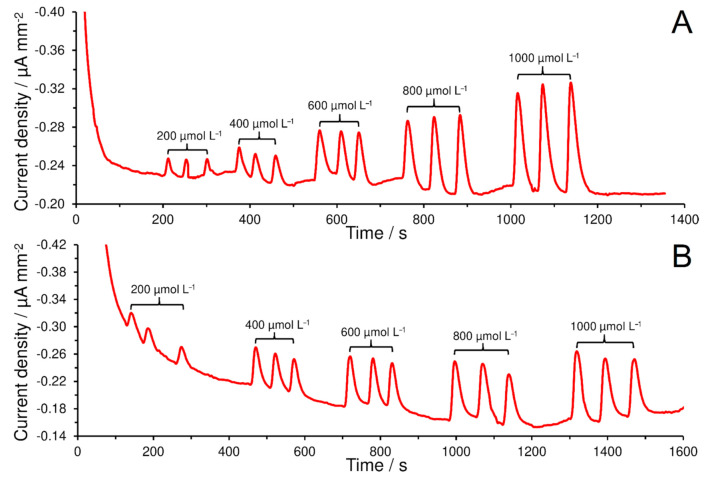
FIA records of dopamine (**A**) and serotonin (**B**) obtained at SPCE/rGO/CBCC-Nafion^®^ at flow rate of 1 mL min^−1^ of 0.1 mol L^−1^ PB containing 0.05 mol L^−1^ KCl and 500 µmol L^−1^ H_2_O_2_ (pH 7.0) and applied potential −0.1 V.

For dopamine, the catalytic hydroxylation may not occur as in the case of monophenol and it is reason, why a higher sensitivity (0.00014 µA mm^−2^ µmol^−1^ L) for dopamine was calculated than in the case of serotonin (0.00009 µA mm^−2^ µmol^−1^ L). For demonstration, the amperometric records obtained at SPCE/rGO/CBCC-Nafion^®^ during calibration measurements of dopamine (A) and serotonin (B) are shown in Figure 6. LOD and LOQ values of 27.8 and 92.7 µmol L^−1^ for dopamine and LOD and LOQ values of 43.8 and 146.0 µmol^−1^ L for serotonin, were calculated using standard deviations of three replicate injections (200 µmol L^−1^ of each neurotransmitter) and slopes of the corresponding calibration curves (sensitivities) characterized by values of determination coefficient higher than 0.9920.

To ensure the precision, the values of repeatability, described by relative standard deviation (7.5 and 6.0% for dopamine and serotonin, respectively) for three replicate measurements, were determined. Due to high mechanical and chemical stability of the CBCC in the Nafion^®^ membrane, the developed amperometric sensors provided a constant current response for time duration for several months.

In the presence of molecular oxygen, a mushroom tyrosinase (EC 1.14.18.1) is able to catalyze the hydroxylation of monophenols in *ortho* position (cresolase activity) and subsequent oxidation of the resulting *ortho*-diphenols to the corresponding *ortho*-quinones and water (catecholase activity) [37]. Unfortunately, biomimetic sensors utilizing the CBCC do not provide required selectivity to be used in the clinical analysis at this stage of development due to similar chemical structure of active site with natural tyrosinase enzyme (two molecules of CBCC connected by oxygen bridges) and absence of protein coats (apoenzymes). Nevertheless, these kind of biomimetic sensors could be integrated into microfluidic separation systems [38].

## 4. Discussion

### 4.1. Tyrosinase Biomimetic Catalyst or Redox Mediator?

To respond to the title question, all of the results shown above suggest that the CBCC has a role as redox mediator in the case of H_2_O_2_ monitoring (electro-sensing). Principally, the copper ion in the CBCC complex can be kept in Cu^+^ form at constant applied potentials lower than 0.0 V. An addition of H_2_O_2_ will cause the chemical oxidation of Cu^+^ to Cu^2+^ form which will be subsequently reduced electrochemically to obtain reduction current response. Generally, it can be predicted that every chemical species capable of chemically oxidizing the monovalent copper ion will provide a reduction current response. Under this assumption, CBCC can only be considered as redox mediator.

**Figure 7:**
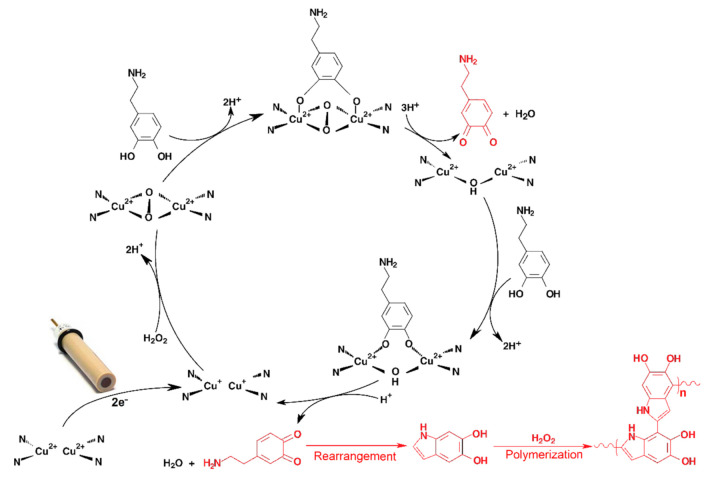
Proposed mechanism for dopamine oxidation catalysed by [Cu(bipy)_2_Cl]Cl∙5H_2_O in the presence of H_2_O_2_.

As shown in Figure 7, the presence of H_2_O_2_ can probably initiate a connection of two electrochemically reduced CBCC molecules by oxygen bridges [26]. The resulting arrangement from above-mentioned interaction is similar to the tyrosinase active site [39].

FIA records, shown in Figure 6, should be therefore considered as a proof that the CBCC represents the tyrosinase biomimetic catalyst, which is able to catalyze the hydroxylation of monophenols, as well as the subsequent oxidation of the resulting ortho diphenols to the corresponding ortho quinones as proposed by Sotomayor et al. [26]. Moreover, the Figure 7 offers an explanation for the formation of polydopamine (PDA), where the dopamine-*o*-quinone (oxidation product of dopamine) is cyclized to form 5,6-dihydroxyindole which subsequently undergoes a polymerization reaction in the presence of H_2_O_2_ [40].

**Figure 8:**
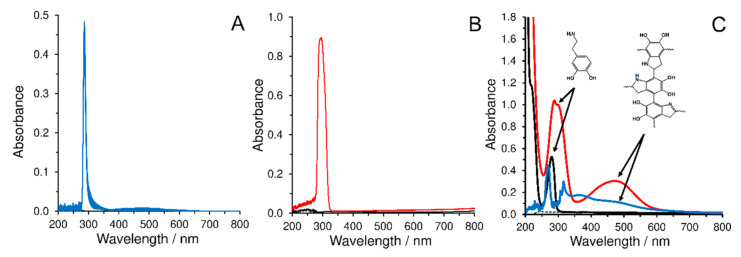
UV-Vis spectra (6 records obtained every 5 min) of 200 µmol L^−1^ dopamine (**A**) and 200 µmol L^−1^ serotonin (**B**) in 0.1 mol L^−1^ PB (pH 7.0) with the presence of 500 µmol L^−1^ H_2_O_2_, the spectrophometric records obtained for comparing the catalytic activity of CBCC in presence of H_2_O_2_ with that of tyrosinase (**C**), 150 µmol L^−1^ dopamine (black) in the presence of 2 µg mL^−^^1^ tyrosinase (red) in the comparison with the record obtained for 150 µmol L^−1^ dopamine in the presence of 300 µmol L^−1^ CBCC and 500 µmol L^−1^ H_2_O_2_ (blue line). Reaction time of 5 min was used in both cases.

The biomimetic activity of the CBCC was studied using the spectrophotometry. First, possible oxidation of the phenolic compounds by H_2_O_2_ was investigated. Two typical representatives of neurotransmitters (200 µmol L^−1^ dopamine and 200 µmol L^−1^ serotonin) were selected to find out whether they undergo the chemical oxidation by 500 µmol L^−1^ hydrogen peroxide. If they are not oxidized to the corresponding quinone forms, no new adsorption bands of their oxidation products will be observed. As shown in Figure 8A,B, dopamine and serotonin provide narrow adsorption bands at 286 and 295 nm, respectively. The effect of H_2_O_2_ presence was investigated every 5 min for half an hour and no significant changes in the obtained spectrums were found. Hence, these neurotransmitters cannot be oxidized by H_2_O_2_ without presence of any catalyst. Furthermore, the catalytic activity of the CBCC toward the dopamine oxidation in presence of H_2_O_2_, was compared with that of the tyrosinase enzyme. As can be seen in Figure 8C, similar to tyrosinase, the adsorption band related to the dopamine oxidation is observed when the CBCC and H_2_O_2_ are present in the dopamine solution that is similar to the addition of tyrosinase into the medium. In fact, these results support the biomimetic activity of the CBCC in presence of the H_2_O_2_ that was also shown by the electrochemical results.

Kinetic studies of the catalytic activity of the CBCC towards neurotransmitters using spectrophotometric measurements were not performed in this study, as might be expected. To be compared with natural tyrosinase enzyme (EC 1.14.18.1), these studies are planned for a number of common neurotransmitters, such as norepinephrine (noradrenaline), epinephrine (adrenaline), noradrenaline, dopamine, L-3,4-dihydroxyphenylalanine (L-DOPA), and serotonin.

### 4.2. Application of [Cu(bipy)_2_Cl]Cl∙5H_2_O Complex-Based Amperometric Sensors

Two types of developed planar amperometric sensors, utilizing the CNMs as measured signal amplifiers and CBCC as redox mediator, could find their application in the fabrication of more complex bioelectroanalytical devices which will be based on amperometric sensing of H_2_O_2_ at 0.0 V vs. Ag/AgCl, especially oxidoreductases amperometric biosensors.

Finally, it can be expected at this stage of development that they can only be used in the analysis of pharmaceutical preparations which usually do not contain any other eventual substrates (neurotransmitters) and potential interferences. However, it is necessary to state that they cannot be directly used as tyrosinase biomimetic sensors in the analysis of clinical samples, especially body fluids, due to low substrate specificity of CBCC in the presence of H_2_O_2_. Although it is not possible to recognize individual neurotransmitters, they could be integrated into hand-held analytical devices as electrochemical detection systems having the upstream separation on monolithic columns [38].

## 5. Conclusions

The present scientific work critically evaluated the possibilities of using the CBCC as tyrosinase biomimetic catalyst and concluded that the studied copper complex could probably replace the unstable natural tyrosinase enzyme (low storage stability at laboratory conditions [41]) in the development of amperometric biomimetic sensors. Unlike the newly synthesized tyrosinase biomimetic catalysts [42,43], the advantage of this cooper complex represents its catalytic activity in the aqueous electrolyte environment which was verified on two representatives of neurotransmitters (dopamine and serotonin). Surprisingly, an oxidation catalytic activity also towards to serotonin was observed which has only one hydroxyl group onto indole ring. However, the CBCC is unable to catalyze the oxidation of these neurotransmitters in the absence of an oxidizing agent (hydrogen peroxide) which complicates itself analysis with derived amperometric sensors, especially in the case of the flow injection analysis.

## Figures and Tables

**Table 1:** Comparison of the prepared amperometric sensors integrated into wall-jet flow cell in FIA of hydrogen peroxide.

Amperometric Sensors	Linear Range (mol L^−1^)	LOD (mol L^−1^)	LOQ (mol L^−1^)
SPCE/CBCC Nafion^®^	1.7 × 10^−4^–4.0 × 10^−3^	4.9 × 10^−5^	1.6 × 10^−4^
SPCE/MWCNTs/CBCC-Nafion^®^	8.0 × 10^−5^–1.5 × 10^−3^	2.2 × 10^−5^	7.5 × 10^−5^
SPCE/rGO/CBCC-Nafion^®^	7.0 × 10^−5^–2.0 × 10^−3^	2.0 × 10^−5^	6.6 × 10^−5^

Note: SPCE; screen-printed carbon electrode (type DRP-C150); CBCC; bis(2,2′-bipyridil)copper(II) chloride complex, MWCNTs; multi-wall carbon nanotubes, rGO; reduced graphene oxide, LOD; limit of detection, and LOQ; limit of quantification.

## Data Availability

Detailed data are available on request to the corresponding author.

## Supplementary Materials

The following are available online at https://www.mdpi.com/1996-1944/14/1/113/s1, Figure S1: Full scan mass positive-ion electrospray mass-spectrum of studied Cu complex; Figure S2: GCEs covered by thin layer of Nafion^®^ membrane with typical rings of [Cu(bipy)_2_Cl]Cl∙5H_2_O complex, Figure S3: Cyclic voltammograms (2 cycles) of 0 (dotted) and 1.0 mmol L^−1^ [Cu(bipy)_2_Cl]Cl∙5H_2_O (solid lines) recorded on bare GCE (A), SPCE (B), GCE/MWCNTs (C), and GCE/rGO (D). All measurements were carried out in 0.1 mol L^−1^ PB (pH 7.5) at potential step of 5 mV and 50 Mv s^−1^; Figure S4: Cyclic voltammograms of 1.0 mmol L^−1^ [Cu(bipy)_2_Cl]Cl∙5H_2_O present in bulk solution at bare GCE (black) and cyclic voltammograms of 0.1 mol L^−1^ PB (pH 7.5) at GCE/CBCC-Nafion^®^ (blue lines) at potential step of 5 mV and scan rates of 20, 40, 60, 80, 100, 140, 180, 220, 260, 300, 400, and 500 mV s^−1^; Figure S5: Amperometric records of five consecutive injections of 100 µmol L^−1^ H_2_O_2_ obtained at SPCE/MWCNTs/CBCC-Nafion^®^ integrated into wall-jet flow cell, 0.1 mol L^−1^ PB (pH 7.0) as flowing carrier solution, flow rate of 1 mL min^−1^, and different applied potentials; Figure S6: FIA record of dopamine (5 injections of 200 µmol L^−1^ every 200 s) obtained at SPCE/MWCNTs/CBCC-Nafion^®^ at flow rate of 1 mL min^−1^ of 0.1 mol L^−1^ PB containing 0.05 mol L^−1^ KCl (pH 7.0) and applied potential −0.1 V.
