# Correction: Sýs et al. Bis(2,2′-bipyridil)Copper(II) Chloride Complex: Tyrosinase Biomimetic Catalyst or Redox Mediator? *Materials* 2021, *14*, 113

**DOI:** 10.3390/ma15134595

**Published:** 2022-06-30

**Authors:** Milan Sýs, Atripan Mukherjee, Granit Jashari, Vojtěch Adam, Amir M. Ashrafi, Miroslav Novák, Lukáš Richtera

**Affiliations:** 1 Department of Analytical Chemistry, Faculty of Chemical Technology, University of Pardubice, Studentská 573, 532 10 Pardubice, Czech Republic; milan.sys@upce.cz (M.S.); granit.jashari@student.upce.cz (G.J.); 2 Department of Chemistry and Biochemistry, Mendel University in Brno, CZ-613 00 Brno, Czech Republic; xmukherj@mendelu.cz (A.M.); vojtech.adam@mendelu.cz (V.A.); ashrafi@mendelu.cz (A.M.A.); 3 Central European Institute of Technology, Brno University of Technology, 612 00 Brno, Czech Republic; 4 Institute of Chemistry and Technology of Macromolecular Materials, Faculty of Chemical Technology, University of Pardubice, Studentská 573, 532 10 Pardubice, Czech Republic; miroslav.novak@upce.cz

## Error in Figure

In the original publication [1], there was a mistake in **Figure 2B** as published. **By mistake, the elemental analysis spectra used in Figure 2A,B are the same**. The corrected **Figure 2B** appears below. The authors apologize for any inconvenience caused and state that the scientific conclusions are unaffected. This correction was approved by the Academic Editor. The original publication has also been updated.

## Figures and Tables

**Figure 2:**
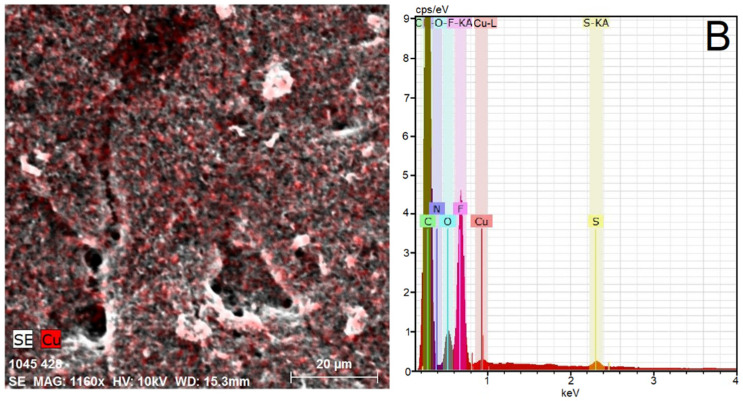
(**B**) Elemental mapping of MWCNTs layers covered by Nafion^®^ membrane containing the [Cu(bipy)_2_Cl]Cl·5H_2_O complex using SEM-EDX spectroscopy.
