# Supplementary material for: Bis(2,2′-bipyridil)Copper(II) Chloride Complex: Tyrosinase Biomimetic Catalyst or Redox Mediator?
Source: Materials (Basel). 2020 Dec 29;14(1):113. doi: 10.3390/ma14010113 (PMC7795177; doi:10.3390/ma14010113)
Supplement: Supplementary file 1 [file materials-14-00113-s001.pdf]

materials

Supplementary material

Bis (2,2'-bipyridil) Copper(II) Chloride Complex: Tyrosinase Biomimetic Catalyst or Redox Mediator?

Milan Sýs [1], Atripan Mukherjee [2,3], Granit Jashari [1], Vojtěch Adam [2,3], Amir M. Ashrafi [2,3], Miroslav Novák [4] and Lukáš Richtera [2,3]*

[1] Department of Analytical Chemistry, Faculty of Chemical Technology, University of Pardubice, Studentská 573, 532 10 Pardubice, Czech Republic; milan.sys@upce.cz (M.S.); granit.jashari@student.upce.cz (G.J.)
[2] Department of Chemistry and Biochemistry, Mendel University in Brno, CZ-613 00 Brno, Czech Republic; xmukherj@mendelu.cz (A.M.); ashrafi@mendelu.cz (A.M.A.);vojtech.adam@mendelu.cz (V.A.); richtera@mendelu.cz (L.R.)
[3] Central European Institute of Technology, Brno University of Technology, 612 00 Brno, Czech Republic; xmukherj@mendelu.cz (A.M.); ashrafi@mendelu.cz (A.M.A.);vojtech.adam@mendelu.cz (V.A.); richtera@mendelu.cz (L.R.)
[4] Institute of Chemistry and Technology of Macromolecular Materials, Faculty of Chemical Technology, University of Pardubice, Studentská 573, 53210, Pardubice, Czech Republic; miroslav.novak@upce.cz (M.N.)
* Correspondence: richtera@mendelu.cz; Tel.: +420-545-133-311

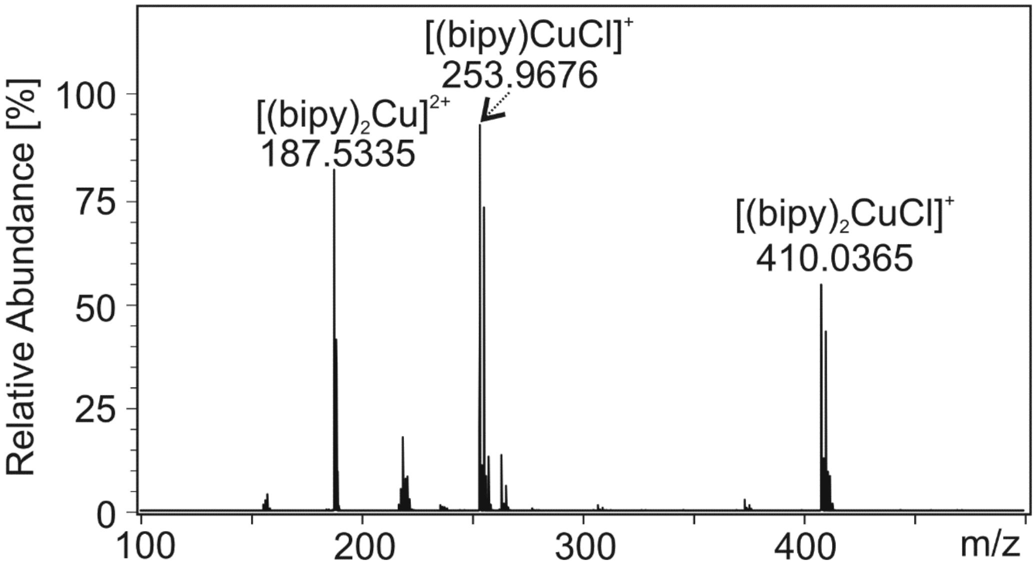

Figure S1. Full scan mass positive-ion electrospray mass spectrum of studied Cu complex.

Positive-ion electrospray ionization mass spectra (ESI-MS) were measured on a hybrid quadrupole time of flight mass analyzer (micrOTOF-Q, Bruker Daltonics, Germany) in the range m/z 100–2000. Samples were dissolved in acetonitrile and analyzed by direct infusion at the flow rate 5 μL/min. The mass spectrometer was used with the following setting of tuning parameters: capillary voltage 3.5 kV, drying temperature 180 °C, the flow rate and pressure of nitrogen were 4 L min^{-1} and 0.4 bar, respectively. The external mass calibration of mass spectrometer was performed with hundred times diluted ESI Tuning Mix (Agilent Technologies, Santa Clara, CA, United States) before individual measurements.

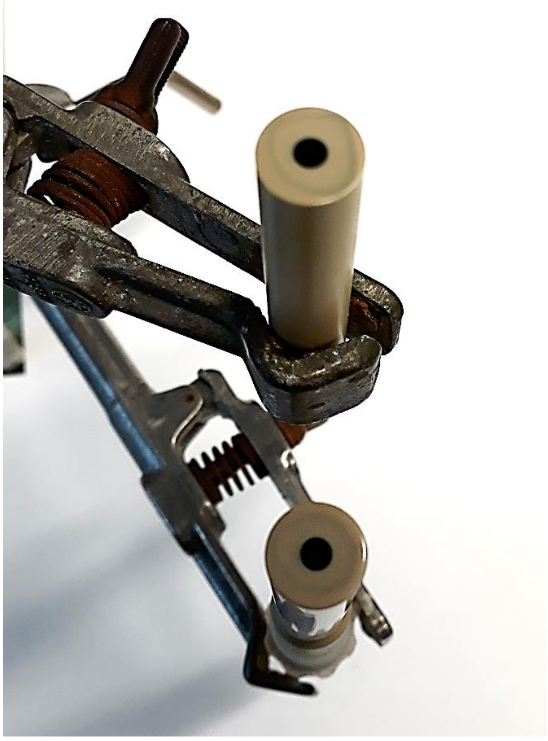

Figure S2. GCEs covered by thin layer of Nafion® membrane with typical rings of [Cu(bipy)$_2$Cl]Cl·5H_2O complex.

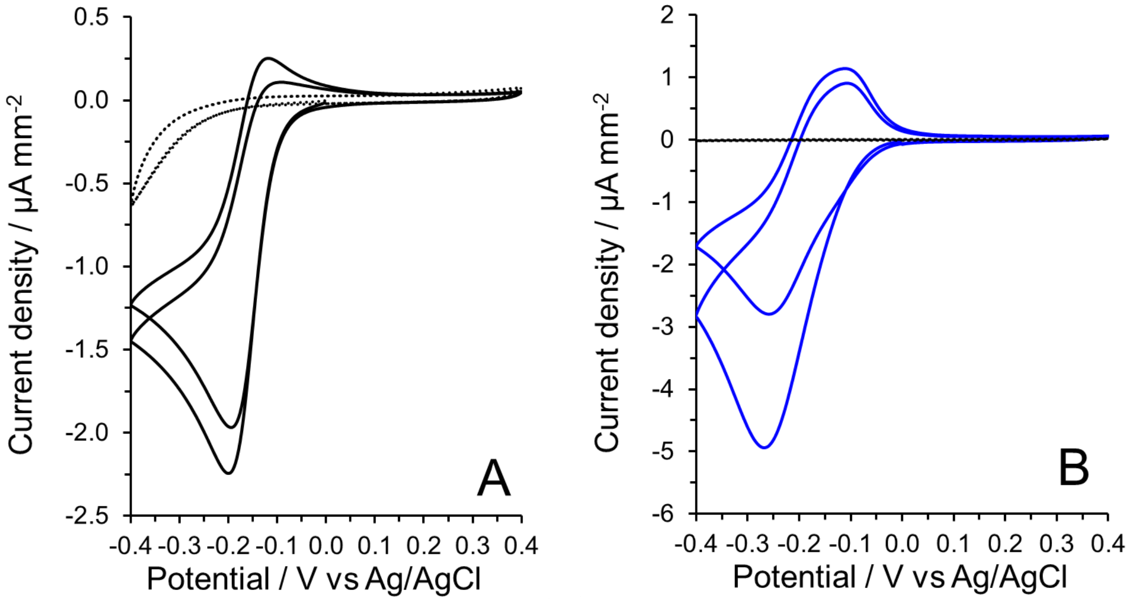

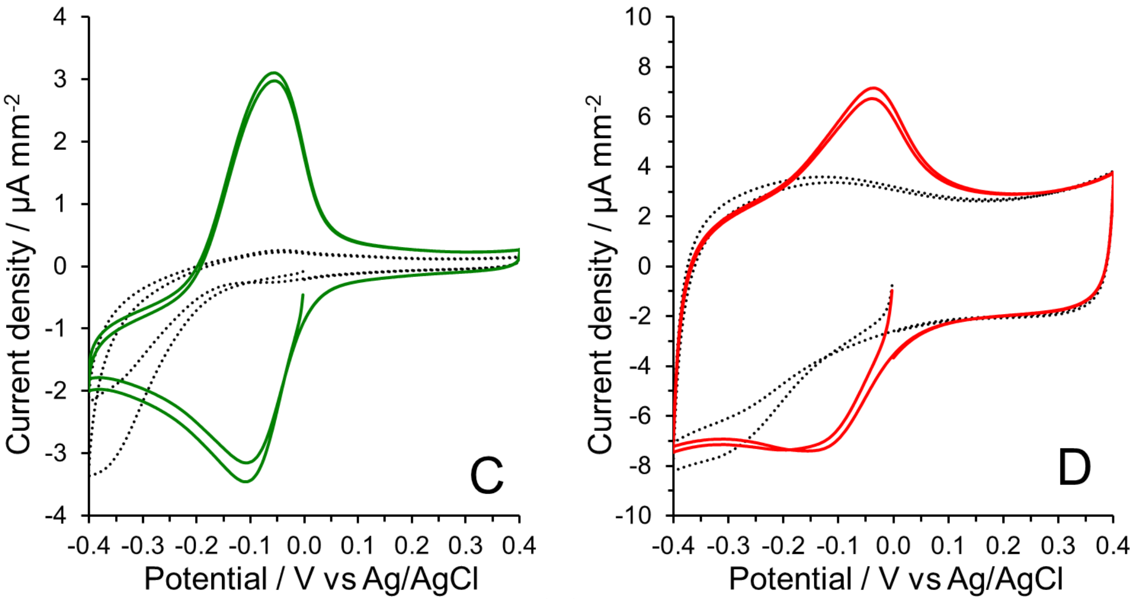

Figure S3. Cyclic voltammograms (2 cycles) of 0 (dotted) and 1.0 mmol L^{-1} $[Cu(bipy)_2Cl]Cl{\cdot}5H_2O$ (solid lines) recorded on bare GCE (**A**), SPCE (**B**), GCE/MWCNTs (**C**), and GCE/rGO (**D**). All measurements were carried out in 0.1 mol L^{-1} PB (pH 7.5) at potential step of 5 mV and 50 Mv s^{-1}.

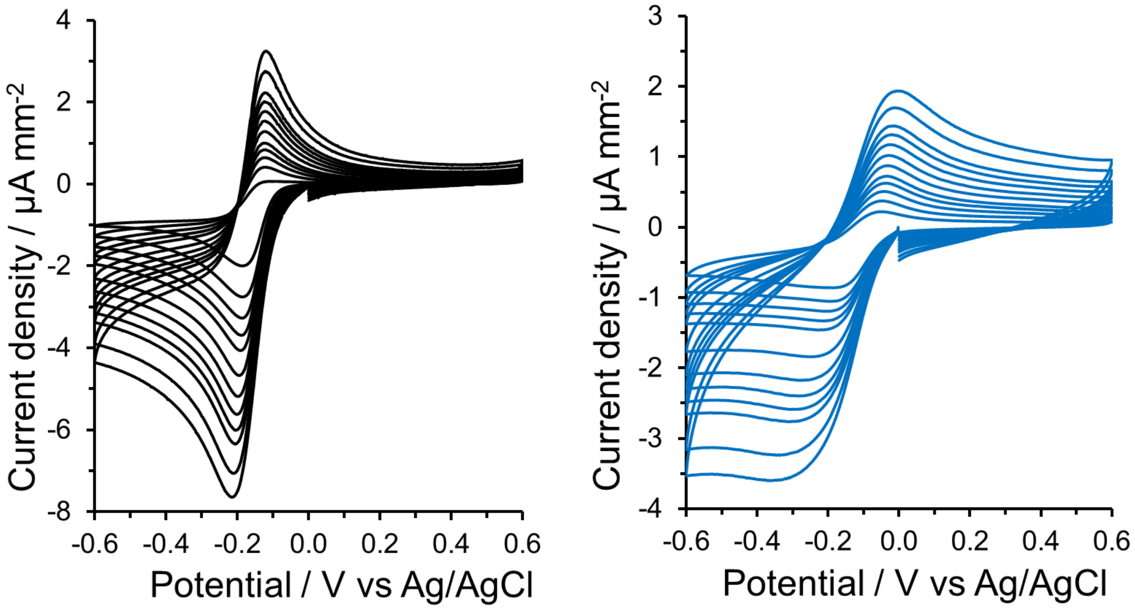

Figure S4. Cyclic voltammograms of 1.0 mmol L^{-1} $[Cu(bipy)_2Cl]Cl{\cdot}5H_2O$ present in bulk solution at bare GCE (**black**) and cyclic voltammograms of 0.1 mol L^{-1} PB (pH 7.5) at GCE/CBCC-Nafion® (**blue lines**) at potential step of 5 mV and scan rates of 20, 40, 60, 80, 100, 140, 180, 220, 260, 300, 400, and 500 mV s^{-1}.

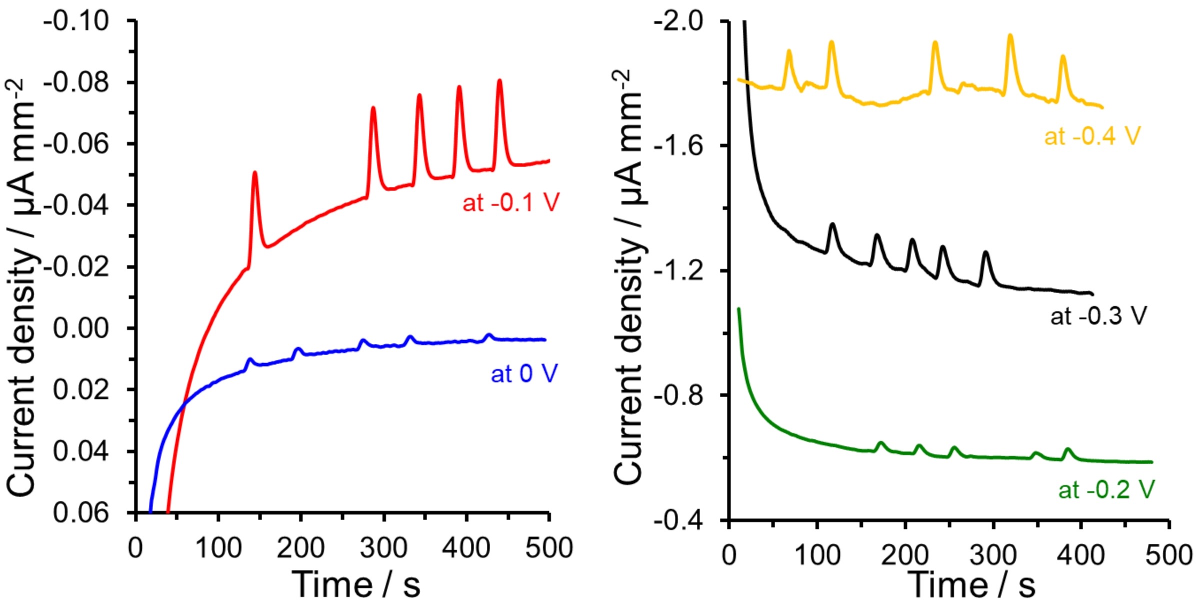

Figure S5. Amperometric records of five consecutive injections of 100 μmol L^{-1} H_2O_2 obtained at SPCE/MWCNTs/CBCC-Nafion® integrated into wall-jet flow cell, 0.1 mol L^{-1} PB (pH 7.0) as flowing carrier solution, flow rate of 1 mL min^{-1}, and different applied potentials.

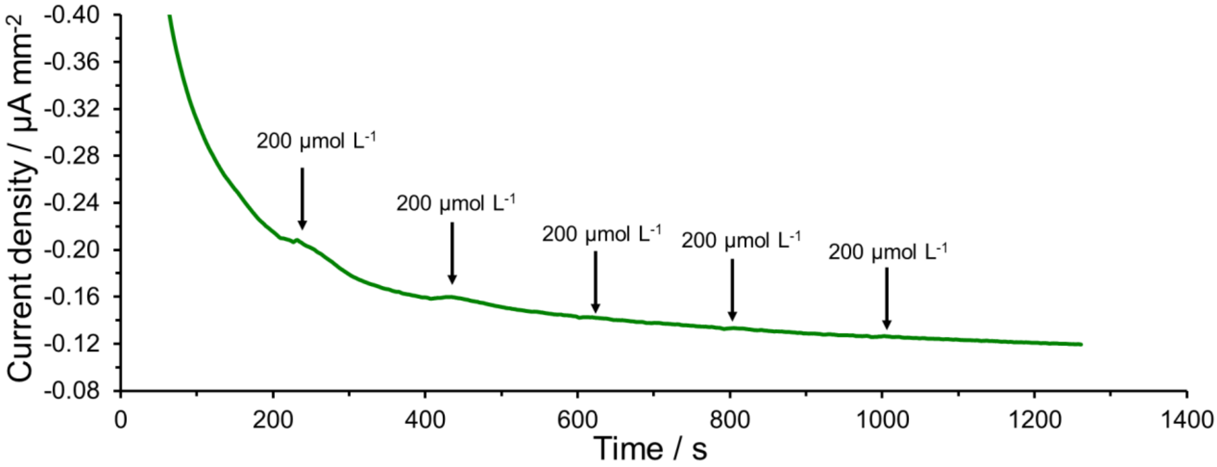

Figure S6. FIA record of dopamine (5 injections of 200 μmol L^{-1} every 200 s) obtained at SPCE/MWCNTs/CBCC-Nafion® at flow rate of 1 mL min^{-1} of 0.1 mol L^{-1} PB containing 0.05 mol L^{-1} KCl (pH 7.0) and applied potential −0.1 V.
